# Novel Milk Substitute Based on Pea, Bean and Sunflower Seeds with Natural Bioactive Stabilisers

**DOI:** 10.3390/plants12122303

**Published:** 2023-06-13

**Authors:** Ewa Kulczyk, Emilia Drozłowska-Sobieraj, Artur Bartkowiak

**Affiliations:** Center of Bioimmobilisation and Innovative Packaging Materials, Faculty of Food Sciences and Fisheries, West Pomeranian University of Technology in Szczecin, Janickiego 35, 71-270 Szczecin, Poland; emilia_drozlowska@zut.edu.pl (E.D.-S.); artur.bartkowiak@zut.edu.pl (A.B.)

**Keywords:** plant-based drinks, legumes, stabilisers, no dairy

## Abstract

The aim of this research was to create a plant-based beverage based on seeds of sunflower (*Helianthus annuus*), pea (*Pisum sativum*) and runner bean (*Phaseolus multiflorus*). The selection of the ingredients was based on the main objective to obtain the nutritional value and sensory characteristics of a formed product similar to cow’s milk. The ingredient proportions were created by comparing the protein, fat and carbohydrate content of seeds versus cow’s milk. Due to the observed low long-term stability of plant-seed-based drinks, a water binding guar gum, a thickener in the form of locust bean gum and gelling citrus amidated pectin containing dextrose were added and evaluated as functional stabilisers. All of the designed and created systems were subjected to selected methods of characterisation of the most important final product properties, such as rheology, colour, emulsion and turbidimetric stability. Rheological analysis confirmed the highest stability of the variant supplemented with 0.5% guar gum. Both stability and colour measurements indicated the positive characteristics of the system supplemented with 0.4% pectin. Finally, the product with 0.5% guar gum was identified as the most distinctive and similar vegetable drink to cow’s milk.

## 1. Introduction

Nowadays, more and more plant-based products are appearing on the market as alternatives to dairy products. Plant-based drinks are produced from cereals, nuts, oil seeds, legume plants and quinoa, which is classified as a pseudo-cereal [1]. There are reports of the development of novel drinks from different varieties of beans and buckwheat, which could form stable emulsions [2]. Mixtures of different raw materials could improve the nutritional properties of various liquid products. Among others, a beverage mixture of almond- and soya-based extracts has been used to achieve more balanced nutritional and valuable sensory profiles [3]. Beverages based on oats, lentils and peas result in a product, which is abundant in the essential amino acids lysine, tyrosine, methionine and cysteine [4]. Currently, a strong focus is observed on novel sources in order to obtain a drink with high concentration of macro- and micronutrients; for example, dried jujube fruit was selected in combination with sunflower seeds [5]. The development of novel milk-like products is intended to limit the development of carcinogenic cells through the presence of selenium [6]. Legume seeds are a valuable source of bioactive compounds, such as phytosterols and isoflavones, which affect the metabolism of the human body [7]. The antioxidant properties of the bean are associated with the presence of tannins, which are mainly found in the external seed coat. Beans are widely known for their superior ability to inhibit the cationic reaction of ABTS^+^ radical formation, which enables the development of special nutritional preparations [8]. Bean-based meal can be both a functional food additive, and it can become the basis for plant-based beverages [9]. Similar to beans, peas are an abundant source of antioxidants. Moreover, the high fibre content can lower blood pressure and improve serum lipid levels, which also reduces inflammation [10]. Oil seeds are a valuable source of unsaturated fatty acids, including linoleic acid, which is involved in metabolism and must be supplied with food. Sunflower seeds contain antioxidants and a number of substances belonging to the flavonoids, i.e., luteolin and quercetin [11].

The reason for the use of legumes and sunflower seeds for the development of novel plant-based drinks could be the high content of essential amino acids [1]. In comparison to cow’s milk, plant sources are more abundant in some amino acids, for example Threonine, Methionine and Histidine (Table 1).

In order to develop a plant-based milk-like beverage, special attention must be paid to the possibility of providing the right composition of essential nutrients, achieving the right texture and sensory qualities. In the case of new plant-based replacements, consumers also look for the appearance, especially colour, homogeneity and the presence of sediment. Plant-based drinks are often different from their animal milk counterpart in these respects because they contain naturally occurring carotene or chlorophyll pigments [16]. On the other hand, in the case of plant dispersions or extracts, the presence of a bean-like aftertaste and earthy smell is an important distinction observed. This phenomenon is characteristic of legumes [1]. Moreover, the main technological problem is stability and the tendency to sediment during storage due to their low thermodynamic stability [17]. Different methods of homogenisation are used to reduce adverse effects [18]. Another method of stabilising emulsion-like beverages is the addition of various polysaccharidic hydrocolloids, such as guar gum, pectin and locust bean gum [19,20]. The main purpose of such composition and process modifications is to prevent detrimental changes of such liquid dispersion systems caused by creaming, sedimentation, coalescence, flocculation, Ostwald ripening or phase separation [3].

The aim of the study was to determine the effect of the addition of selected hydrocolloids (guar gum, pectin, locust bean gum) on the stability and physical parameters of the created cow-milk-like plant-based beverage based on sunflower seeds, peas and beans as the main protein and saccharide substances and sources of bioactive compounds. The following beverage variants were analysed: pectin 0.4% (P 0.4%); pectin hydrated 0.4% (PH 0.4%); guar gum 0.5% (GG 0.5%); guar gum hydrated 0.5% (GGH 0.5%); locust bean gum 0.5% (LBG 0.5%); locust bean gum hydrated 0.5% (LBGH 0.5%). The possibility of using simple methods to create a plant-based beverage was also indicated, considering the basic analyses that characterise the resulting product, i.e., rheological and colour changes in the CIE Lab scale of obtained beverages were analysed during two weeks of storage under refrigerated conditions (7 °C). In addition, the turbidimetric stability of the beverages was determined.

## 2. Results

### 2.1. Measurement of Seeds’ Water Loss

The highest water loss was observed for bean seeds (Table 2). In contrast, the lowest water loss was identified in sunflower seeds.

### 2.2. Rheological Analysis

Rheological analysis indicated a significant effect of pre-hydration of the stabilisers, consisting of dispersing the stabiliser in water and leaving it at 7 °C for 24 h. An interpretation of the results was obtained for prior samples without hydration.

The GG 0.5% and LBG 0.5% variants showed an increase in viscosity of the beverage after a two-week incubation (Table 3). LBG 0.5% indicated very high differences, in stark distinction from the other variants. However, the solution supplemented with P 0.4% showed a decrease in viscosity of 12 [mPa × s] in contrast to the others.

**Figure 1:**
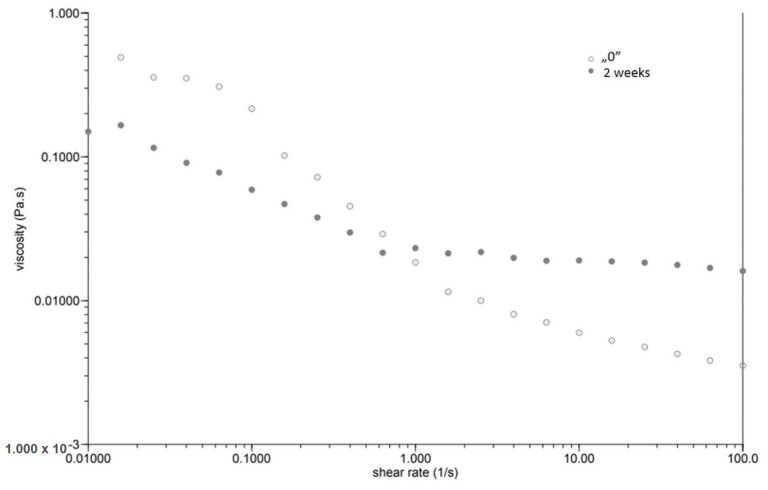
Dependence of viscosity presented as a function of shear rate for a beverage supplemented with 0.4% pectin.

**Figure 2:**
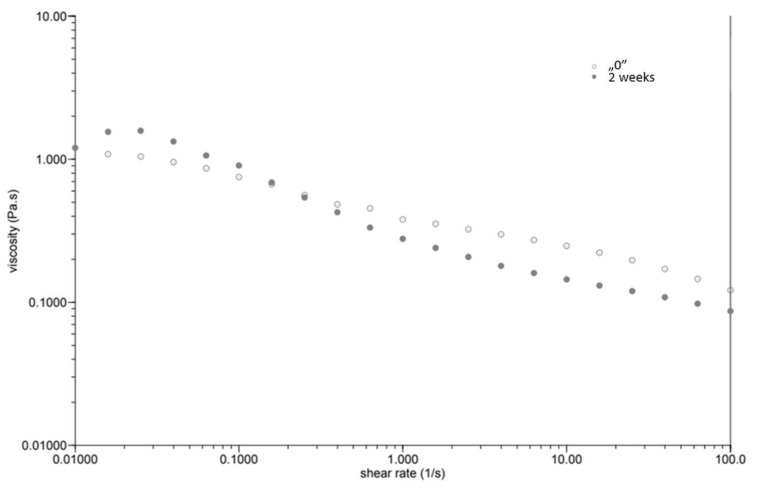
Dependence of viscosity presented as a function of shear rate for a beverage supplemented with 0.5% guar gum.

**Figure 3:**
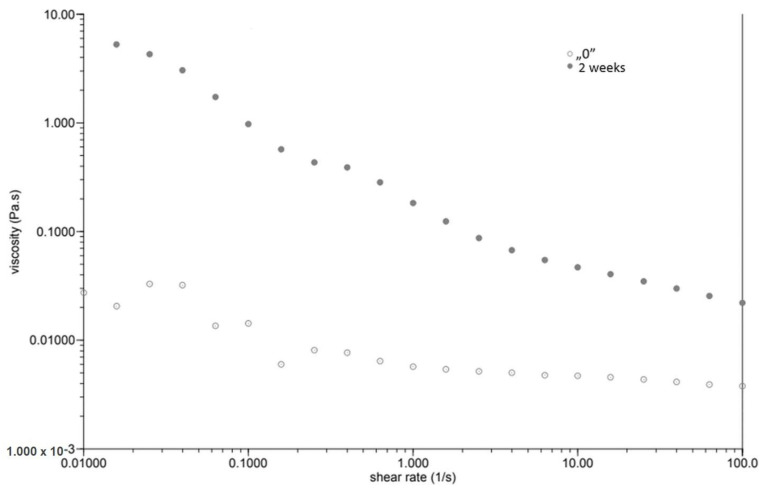
Dependence of viscosity presented as a function of shear rate for a beverage supplemented with 0.5% locust bean gum.

At the same time, as the shear rate increased, the viscosity of the “0” sample decreased (Figure 1). The sample behaved similarly after incubation, while the viscosity stabilised once the shear rate increased above 1.0 [1/s]. The GG 0.5% variant (Figure 2), in contrast to P 0.4%, showed a lower initial viscosity on the day of preparation than after incubation. Both measurements on day “0” before and after incubation showed a decrease in viscosity with increasing shear rate. The addition of locust bean gum in the LBG 0.5% beverage (Figure 3) on day “0” affected the low shear rate resistance of the beverage, as indicated by both decreases and increases in viscosity. After a two-week incubation, the viscosity of the drink decreased as the shear rate increased.

### 2.3. Colour Measurement in CIE Lab Space

The highest level of the L* parameter for the variants was obtained for GG 0.5%; consequently, in terms of brightness, it was most similar to milk with 0.5 [%] fat (M 0.5%) (Table 4). The previous hydration (GGH 0.5%) did not result in a significant decrease in this parameter. The pectin-infused drink also showed high levels of lightness. The lowest value was obtained for LBG 0.5%, indicating the low colour similarity to cow’s milk. The a* and b* parameters for M 0.5% differed from those obtained in the resulting drinks.

The smallest colour differences during the two-week storage of the beverages were determined for P 0.5% (Table 5). Slight differences were also observed for GG 0.5%, GGH 0.5% and LBH 0.5%. A large difference was only noted for LBGH 0.5%, which does not favour the consumer perception of the product.

### 2.4. Emulsion Stability Index EI

**Figure 4:**
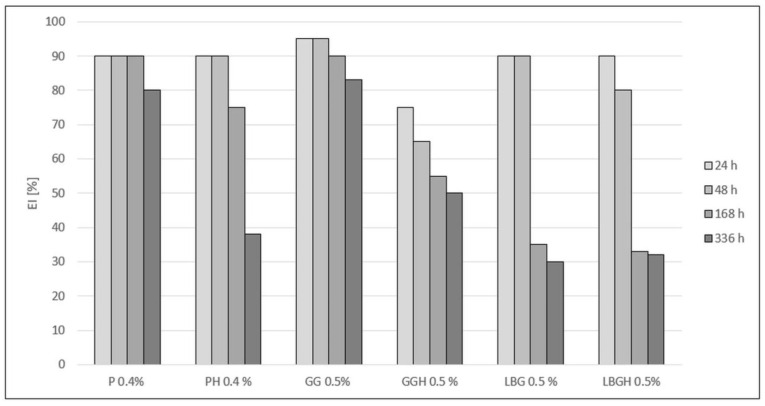
Comparison of the EI stability index for the created variants after different storage times. P 0.4%—Pectin 0.4%; PH 0.4%—Pectin hydrated 0.4%; GG—Guar gum 0.5%; GGH—Guar gum hydrated 0.5%; LBG 0.5%—Locust bean gum 0.5%; LBGH 0.5%—Locus bean gum hydrated 0.5%.

All variants supplemented with hydrated stabilisers showed lower emulsion stability compared with variants where the stabilisers were not hydrated (Figure 4). Only the P 0.4% variant showed stability at the same level for 168 h (1 week). The drink with 0.5% GG showed a decrease in stability of approximately 5% after 168 h and after 336 h (2 weeks). The lowest stability was observed for LBG 0.5% and LBGH 0.5% (more than half).

### 2.5. Turbidimetric Stability Analysis

The non-hydrated variants showed a higher decrease in the R-parameter (at *p* < 0.05) over time than the hydrated variants (Table 6). The drink with PH 0.4% showed the least change in particle size during storage within a range of change of 0.587–0.630. A decrease in the R-value after 10 min was observed in all variants. Only the LBG 0.5% remained at the same level. After 336 h (2 weeks), the highest R-difference was determined for GGH 0.5%.

After 10 min in the PH 0.4% variant, the O-index was on the same level. In contrast, its value decreased in the other cases. After 24 h, an increase in the O-index was observed in the P 0.4% and GG 0.5% beverages, in contrast to beverages with a hydrated stabiliser. The highest differences after 336 h (2 weeks) were observed for versions with a hydrated stabiliser.

## 3. Discussion

Plant-based aqueous beverages could be produced by wet milling (after cooking) or dry milling (after drying the seeds) the ingredients. The next step is separation of the particulates [21]. Before milling, the seeds were additionally subjected to drying at mild conditions at 40 °C for 5 h to avoid agglomeration when exposed to moisture during the milling process. Only in the case of sunflower seeds was it possible to reduce this effect. Water loss during drying is shown in Table 1. The drying temperature of the seeds is usually in the range of 40–70 °C [22]. Drying may cause damage to the seed coat and thermal denaturation of proteins. The use of drying temperatures ≥ 70 °C increases seed damage. Therefore, in this experiment, a stable low drying temperature of 40 °C was selected to reduce the negative effect of hot air on the chemical stability and high nutrient quality of the seeds [23].

The grinding process was carried out until a particle size in the range of 100–500 micrometres was achieved. In order not to overheat the product, seeds were crushed: 1 min for beans, 2 min for peas and 0.5 min for sunflower. Further on in the course of the experiment, 0.1% oil was added to obtain a fatty phase in the drink and selected stabilisers (4.2). A low oil addition was used because a higher content caused excessive foaming of the beverage during homogenisation, resulting in the need for early termination of the process and a lack of adequate homogeneity of the beverage. The addition of individual stabilisers was indicated in earlier studies to be of prime importance in the creation of a soya beverage. The authors have analysed different concentrations of various stabilisers, such as guar gum and locus bean gum, which, based on their analyses, made it possible to select the most preferred variants [24]. The use of roasted locust bean gum at a final concentration of 0.5 wt.% made it possible to produce a drink similar in consistency and colour to cocoa drink due to the characteristic colour of the stabiliser. The inclusion of other polysaccharides in the present study was aimed at obtaining a colour similar to cow’s milk. After each preparation step, the stability of the systems was checked to look for the most preferred option.

Right after preparation, the P 0.4% drink showed a slightly higher initial viscosity than samples after two weeks of incubation (Table 3). The GG 0.5% beverage (Figure 2) showed a low difference in initial viscosity before and after storage due to the ability of guar gum to rapidly hydrate [25], in contrast to the beverage with pectin (Figure 1). The significant difference in the initial viscosity of the LBG 0.5% beverage is due to the time needed to reach maximum viscosity, which is approximately 2 h [26]. All variants show an image characteristic of a non-Newtonian liquid with pseudo-plastic character. A deviation can be seen in the LBG 0.5% variant at day “0”, where the viscosity versus shear rate curve approaches a straight line, which characterises a Newtonian liquid. At higher shear rates, the molecular entanglements may be broken and new ones formed, allowing viscosity to remain constant [26].

Colour is one of the basic but sometimes most important parameters in the quality assessment of food products and raw materials. The aim of the measurements was to estimate the changes between plant-based beverages and cow’s milk. Due to this assumption, the colour parameters were measured for 0.5 [%] fat milk (Mlekovita, Wysokie Mazowieckie, Poland) and compared with plant-based drinks (Table 4). The M 0.5% product has the highest brightness due to the reflection and scattering of light by the dispersed fat balls, calcium caseinate and calcium phosphate [27]. The lightness parameter is also affected by the size of the fat particles and their number. An increase in fat droplets results in greater light scattering [28]. In formulated plant-based drinks, fat globules are also formed due to the addition of sunflower oil, while the presence of natural pigments present in the seeds causes some divergences in the measured parameters [18]. These differences are also due to the high variety of ingredients and selected methods of preparing plant-based drinks [29]. The parameter a* for M 0.5% has a negative value, which indicates the direction of green. In the case of the vegetable beverages obtained, the opposite situation occurs, and the observed high (+) a* parameter points in the direction of red. The closest variant with respect to the parameters analysed is the beverage supplemented with guar gum, which has the brightest colour compared to the other stabilisers used.

The total colour difference after a two-week incubation (ΔE*) in most samples was defined as minimal or with perceived differences (Table 5). Similar results were obtained by Jeske et al. (2019) investigating the colour stability of a lentil drink after 21 days. The minimal variation in colour change of the beverage after storage may be indicative of the absence of coalescence, causing the formation of larger droplets, which might lead to less effective scattering of light, which results in a darker colour of the solution [30].

Analysing the EI index, the P 0.4% sample showed stability at the same level after one week of storage, followed by a 10% decrease in the second week of the storage test (Figure 4). In contrast, PH 0.4% remained stable for only 48 h, which may indicate the absence of sufficient push-off forces once the dispersed particles interact with pectin, and the cream phase first tends to coalescence and then separate into two phases. Pectin can also stabilise a beverage by forming viscous gel networks in which oil droplets are dispersed, while the long-term inability to entrap fat globules results in phase separation of the drink and a loss of stability [31]. High EI values were shown by the GG 0.5% variant, which had a stability in the range of 80–91% over the whole storage period. The high stability of the 0.5% GG sample may be due to the swelling properties of guar gum in the aqueous phase and formation of strong hydrogen bonds with other polysaccharidic molecules [25]. Ercelebi EA et al. (2009) also reported a beneficial effect of guar gum on the stabilisation of chicken egg white, which indicates a wide range of applications of this polysaccharide for the stabilisation of colloidal systems that include both plant and animal proteins [32]. A different situation can be observed with the LBG 0.5% variant. A rapid decrease of 47% occurred after only one week of incubation, which was the same as the appearance of serum and large dispersed phase particles, indicating a coalescence effect. However, among the galactomannans (including guar gum), locust bean gum has the lowest viscosity, which results in lower viscosity and stabilising properties of the various aqueous systems [26]. On the other hand, the use of the stabiliser in the emulsion made from bean protein showed a beneficial effect on stability [33]. The presence of extracted seed proteins also helps stabilise the beverages. The multi-polarity of the proteins allows them to interact with water, lipids and other components of the drink. Water binding of GG 0.5% occurs through the formation of a gel structure and holding the water molecules in the system [34]. Stabilisers that were hydrated bound the water molecules and then did not completely stabilise the drinks when introduced into the system. In addition, too much water prevented the proteins from binding, which resulted in a process of destabilisation and phase separation of the beverage due to thermodynamic incompatibilities [35].

A decrease in the R-index after 10 min (Table 6) indicates the occurrence of gravitational separation, consisting of the downward movement (sedimentation) of beverage particles with a greater density than the surrounding liquid. During beverage storage, the R-index shows instability through increases and decreases in its values. An increase in the R-index may indicate the formation of aggregates of molecules [36]. In addition, molecules can migrate from the sedimentation layer into the dispersed phase of the solution and then drop back down again, as seen in the R-index reading [30]. Dłużewska et al. (2005) indicated an increase in particle size in beverage emulsions after 12 days of storage with and without the addition of a stabiliser, suggesting that changes related to its instability resulted in the occurrence of the aforementioned phenomena [36]. The P 0.4% variant had a higher R-index than PH 0.4%, which may indicate the formation of a gel network to hold the suspension particles [28]. The high R-index of beverages supplemented with guar gum is related to swelling and the formation of a colloidal dispersion [25]. Variants of beverages with hydrated stabilisers have a lower particle size due to earlier water binding, resulting in a less stable product. Physical instability effects may make a difference to the R-index [30].

The degree of turbidity (O) changed during beverage storage, which was also observed in the R-index (Table 7). The highest degree of turbidity was found for the GG 0.5% variant. The O-index reaches its highest value after 24 h of incubation because, only after this time, the stabiliser is completely dissolved [25]. Changes in the O-index are associated with particle migration related to the lack of a stable colloidal structure in beverages. Furthermore, after a period of two weeks, there was an increase in the O-index in each of the variants, with the exception of PH 0.4%. The increase in the O-index confirms that stabilisers may be a factor in turbidity. Similar results were obtained by Dłużewska et al. (2008), which also showed an increase in turbidity in a beverage emulsion after 12 days of incubation. 

## 4. Materials and Methods

### 4.1. Materials

The shelled seeds of sunflower (*Helianthus annuus*) from Makar (Katowice, Poland), pea (*Pisum sativum*) in the form of shelled halves and runner beans (*Phaseolus multiflorus*) from Plony Natury (Kostrzyn, Poland) were used to create the vegetable drink. Guar gum (E412), locust bean gum (E410) (BioPlanet, Leszno, Poland) and citrus amidated pectin (E440) containing dextrose (Targrochfil, Zakliczyn, Poland) were used as stabilisers. In addition, 0.1% sunflower oil (Wielkopolski, Szamotuły, Edible Oils Limited) and demineralised water were added as an aqueous phase.

### 4.2. Preparation of the Plant-Based Beverage

A mechanical grinder IKA A11 basic (IKA, Darmstadt, Germany) was used to grind the seeds. The ground seeds were placed in screw-top containers to protect them from moisture. The proportion of seed addition was determined by comparing the carbohydrate, protein and fat content with cow’s milk (Table 8). The composition of the final recipe of the proposed vegetable beverage is shown in Table 9. An appropriate amount of water was added, and the seeds were blended in a blender (Philips HR 2000/70, Amsterdam, The Netherlands) and homogenised at a rotational speed of approximately 15,000 rpm in a knife homogeniser Unidrive 1000 (CAT, Ballrechten-Dottingen, Germany). The final aqueous suspension was filtered through a 3 mm cotton mesh sieve, and the resulting filtrate was used for further testing and modification.

Stabilising agents in the form of aqueous solutions were added directly to the drink and after pre-hydration (suspended in water and incubated at 7 °C/24 h), creating the following drink variants:

- P: 0.4% of Pectin 0.4%;
- PH: 0.4% of Pectin hydrated 0.4%;
- GG: 0.5% of Guar gum 0.5%;
- GGH: 0.5% of Guar gum hydrated 0.5%;
- LBG: 0.5% of Locust bean gum 0.5%;
- LBGH: 0.5% of Locust bean gum hydrated 0.5%.

In order to hydrate each of the polysaccharidic stabilisers (P, GG or LBG) after solubilisation in deionised water, they were incubated at 7 °C for 24 h to produce a viscous solution with improved stabilising properties [25]. After the addition of stabilisers to the filtered suspension, each of the prepared samples was homogenised using a Unidrive 1000 knife homogeniser (CAT, Ballrechten-Dottingen, Germany) at 8500 rpm for 1 min. Then, such systems, as prepared, were pasteurised for 15 min at 60 °C and then cooled down to 20 °C. The final step involved homogenisation of the 100 mL dispersion using a Unidrive 1000 knife homogeniser (CAT, Ballrechten-Dottingen, Germany) at 8500 rpm for 5 min. The created beverages were stored for two weeks under refrigeration (7 °C).

### 4.3. Measurement of Water Loss Contained in Seeds

The seeds were positioned on aluminium foil and then dried in a dryer (Avantgarde.Line, Tuttlingen, Germany) with natural air circulation at 40 °C for 5 h.

### 4.4. Rheological Analysis

Rheological characterisation of the final beverage emulsions was performed using an AR-G2 rotational rheometer (TA Instruments, New Castle, DE, USA) with a 60 mm diameter cone-plate geometry system at 20 °C. The measurements were conducted using the selected dynamic mode in a shear rate range of 25–100 [1/s] immediately after the creation of the beverage and after two weeks of storage at a temperature of 4 °C.

### 4.5. Colour Measurement in CIE Lab Space

The colour of all samples was measured on the day of preparation and after two weeks with a Konica Minolta CR-5 colourimeter (Konica Minolta, Osaka, Japan). The following values—L* value (white 100/black 0), a* value (redness/greenness) and b* value (yellowness/blueness)—were used to calculate the total colour difference (ΔE*) using the following equation:

ΔE* = [(ΔL*)^2^ + (Δa*)^2^ + (Δb*)^2^]^1/2^

For a final interpretation of the results, the following ΔE* scale was applied: 

ΔE* < 1—differences not perceptible;

1 ≤ ΔE* < 2—minimum differences;

2 ≤ ΔE* < 3—poorly perceived;

3 ≤ ΔE* < 5—perceptible differences;

5 ≤ ΔE* <12—large difference;

≥12—different colour [43].

### 4.6. Emulsion Stability Index EI

The prepared samples were placed in 30 mL Egzert tubes and incubated at 7 °C for two weeks. The stability of the emulsion was determined using the following formula [44]:

$$EI=\frac{volume\;of\;the\;cream\;phase}{total\;volume}\times 100\%$$

### 4.7. Turbidimetric Stability Analysis

The stability of the beverage emulsion was measured using the turbidimetric method by determining the absorbance of diluted samples at a ratio of 1:1000. The measurements were taken at wavelengths of 400, 600, 800 nm, using an Evolution 2020 UV-VIS spectrophotometer (Thermo Fisher Scientific Inc., Waltham, MA, USA). The ratio of absorbance at 800 and 400 nm was attributed to the particle size index (R), and the absorbance at 600 nm to the degree of turbidity (O) [36].

### 4.8. Statistical Analysis

All the experiments were repeated three times. All the data were expressed as mean value ± standard deviation (SD). Statistical significance was tested by an analysis of variance (one- and two-way ANOVA) followed by Fisher’s NIR test. All analyses were performed using Statistica software version 13.03 (StatSoft Polska, Kraków, Poland). The compared values were considered significantly different at *p* < 0.05.

## 5. Conclusions

In all the evaluated variants, the process of pre-suspending natural stabilisers in deionised water and incubation for 24 h at a refrigeration temperature prevented the formation of stable plant drinks. As part of beverage preparation, stabilisers should be added directly to the system. The dependence of viscosity as a function of shear rate showed differences in the course of the experiment for systems after a two-week incubation, indicating a slow stabilisation of interaction between the beverage ingredients. Table 10 provides a summary of the parameters for comparing the beverage options.

The most favourable variant, allowing stabilisation and a high similarity to cow’s milk, is the drink supplemented with 0.5% guar gum. The addition of amidated pectin also indicates the possibility of a product with positive characteristics. However, rheological analysis showed a higher stability of the system supplemented with guar gum. The use of locust bean gum makes it possible to produce a plant product similar to cocoa due to the dark colour of the stabiliser powder. However, this specific option requires further analysis and research to finally achieve a stable product. Regarding nutrient content, Table 8 indicates the theoretical content. The indications are 3.17, 2.18, 3.24 g/100 mL of carbohydrates, proteins and fats, respectively, for the beverage created. In the future, a full chemical, biochemical and sensory analysis of the most preferred variant would have to be carried out in order to match the product with consumer expectations. It is also worth verifying the actual nutrient content of the plant-based drink for a final comparison with cow’s milk.

## Figures and Tables

**Table 1:** Content of essential amino acids [g/100g].

Amino Acid	Thr	Val	Met	Ile	Leu	Phe	Lys	His	Arg	References
Beans	0.87–0.97	1.19–1.31	0.05–0.08	0.92–0.97	2.16–2.26	1.00–1.12	1.41–1.61	0.53–0.58	2.23–2.56	[12]
Peas	0.64–0.88	0.74–1.06	0.03–0.05	0.60–0.78	1.42–1.83	0.87–1.00	1.04–1.48	0.34–0.42	1.26–1.95	
Sunflower	0.70–0.82	0.95–1.24	0.40–0.50	0.78–0.99	1.26–1.53	0.88–1.18	0.50–0.61	0.34–0.42	1.26–1.95	[13]
Cow’s milk	0.12–0.15	0.15–0.20	0.07–0.09	0.13–0.17	0.27–0.33	0.13–0.17	0.25–0.27	0.08–0.09	0.10–0.11	[14,15]

Thr—Threonine; Val—Valine; Met—Methionine; Ile—Isoleucine; Leu—Leucine; Phe—Phenylalanine; Lys—Lysine; His—Histidine; Arg—Arginine.

**Table 2:** Seed water loss during drying at 40 °C for 5 h.

Seeds	% Water Loss
Sunflower	2.0
Beans	7.5
Peas	4.0

**Table 3:** Viscosity of samples on the day of preparation and after two weeks of storage.

Incubation	P 0.4%	GG 0.5%	LBG 0.5%
[mPa × s]			
“0”	161	1083	27
2 weeks	149	1198	5258

**Table 4:** Measurement of L*, a*, b* parameters for the variants analysed and milk (measured on the day—initial day).

Variant	L*	a*	b*
M 0.5%	88.67 ± 0.05 ^a^	−3.60 ± 0.01 ^a^	7.78 ± 0.01 ^a^
P 0.4%	73.40 ± 0.07 ^b^	1.00 ± 0.01 ^b^	10.47 ± 0.01 ^b^
PH 0.4%	73.68 ± 0.07 ^c^	0.89 ± 0.01 ^c^	10.93 ± 0.10 ^c^
GG 0.5%	75.63 ± 0.04 ^d^	1.16 ± 0.01 ^d^	11.58 ± 0.00 ^d^
GGH 0.5%	75.20 ± 0.10 ^e^	1.05 ± 0.01 ^e^	12.16 ± 0.12 ^e^
LBG 0.5%	64.73 ± 0.35 ^f^	5.07 ± 0.15 ^f^	16.30 ± 0.12 ^f^
LBGH 0.5%	70.43 ± 0.12 ^g^	2.12 ± 0.04 ^g^	12.38 ± 0.12 ^g^

Values are mean ± standard deviation from three repeated determinations. ^a, b, c, d, e, f, g^—means with different lowercase letters in the same column are significantly different at *p* < 0.05. M 0.5% —milk with 0.5% fat; P 0.4%—Pectin 0.4%; PH 0.4%—Pectin hydrated 0.4%; GG—Guar gum 0.5%; GGH—Guar gum hydrated 0.5%; LBG 0.5%—Locust bean gum 0.5%; LBGH 0.5%—Locus bean gum hydrated 0.5%; L*: lightness; a*: redness/greenness; b*: yellowness/blueness.

**Table 5:** The evaluation of ΔE* index.

Variant	ΔE*	Interpretation
P 0.4%	1.20	Minimum differences
PH 0.4%	3.84	Perceived differences
GG 0.5%	1.59	Minimum differences
GGH 0.5%	1.33	Minimum differences
LBG 0.5%	1.85	Minimum differences
LBGH 0.5%	8.70	Large difference

P 0.4%—Pectin 0.4%; PH 0.4%—Pectin hydrated 0.4%; GG—Guar gum 0.5%; GGH—Guar gum hydrated 0.5%; LBG 0.5%—Locust bean gum 0.5%; LBGH 0.5%—Locus bean gum hydrated 0.5%; ΔE*: total color difference.

**Table 6:** Particle size (R) in the beverage variants tested.

Variant	„0”	10 min	24 h	48 h	168 h	336 h
P 0.4%	0.739 ± 0.001 ^Aad^	0.737 ± 0.001 ^Aa^	0.762 ± 0.003 ^Ca^	0.740 ± 0.001 ^Aa^	0.737 ± 0.002 ^Aa^	0.709 ± 0.001 ^Fa^
PH 0.4%	0.616 ± 0.002 ^Ab^	0.614 ± 0.000 ^Bb^	0.612 ± 0.036 ^Bb^	0.630 ± 0.002 ^Bb^	0.610 ± 0.001 ^Bb^	0.587 ± 0.001 ^Bb^
GG 0.5%	0.689 ± 0.001 ^Ac^	0.688 ± 0.000 ^Ac^	0.747 ± 0.001 ^Cc^	0.703 ± 0.003 ^Ac^	0.716 ± 0.001 ^Ac^	0.670 ± 0.001 ^Fc^
GGH 0.5%	0.908 ± 0.002 ^Aa^	0.706 ± 0.000 ^Ad^	0.694 ± 0.000 ^Ab^	0.678 ± 0.001 ^Ad^	0.708 ± 0.001 ^Ad^	0.684 ± 0.007 ^Aa^
LBG 0.5%	0.668 ± 0.002 ^Aad^	0.668 ± 0.000 ^Aae^	0.664 ± 0.000 ^Cc^	0.686 ± 0.001 ^Ce^	0.630 ± 0.001 ^Ee^	0.600 ± 0.007 ^Fd^
LBGH 0.5%	0.596 ± 0.001 ^Ad^	0.584 ± 0.001 ^ABe^	0.621 ± 0.002 ^Ae^	0.619 ± 0.001 ^Ba^	0.563 ± 0.010 ^Ee^	0.541 ± 0.003 ^Fe^

Values are mean ± standard deviation from three repeated determinations. ^a, b, c, d, e^—means with different lowercase letters in the same column are significantly different at *p* < 0.05. ^A, B, C, E, F^—means with different uppercase letters in the same row are significantly different at *p* < 0.05. P 0.4%—Pectin 0.4%; PH 0.4%—Pectin hydrated 0.4%; GG—Guar gum 0.5%; GGH—Guar gum hydrated 0.5%; LBG 0.5%—Locust bean gum 0.5%; LBGH 0.5%—Locus bean gum hydrated 0.5%.

**Table 7:** Degree of turbidity (O) in the beverage variants tested.

Variant	„0”	10 min	24 h	48 h	168 h	336 h
P 0.4%	0.489 ± 0.001 ^Aa^	0.482 ± 0.000 ^Aa^	0.673 ± 0.001 ^Ca^	0.803 ± 0.001 ^Da^	0.620 ± 0.001 ^Aa^	0.381 ± 0.001 ^Fa^
PH 0.4%	0.361 ± 0.002 ^Ab^	0.361 ± 0.001 ^Bb^	0.286 ± 0.001 ^Bb^	0.597 ± 0.004 ^Bb^	0.647 ± 0.003 ^Bb^	0.700 ± 0.002 ^Ab^
GG 0.5%	1.023 ± 0.001 ^Ac^	1.004 ± 0.001 ^Ac^	1.376 ± 0.001 ^Cc^	1.083 ± 0.001 ^Ac^	1.168 ± 0.001 ^Ac^	0.899 ± 0.001 ^Fc^
GGH 0.5%	0.908 ± 0.002 ^Ad^	0.878 ± 0.001 ^Ad^	0.867 ± 0.001 ^Ad^	0.491 ± 0.002 ^Ad^	0.639 ± 0.002 ^Ad^	0.488 ± 0.008 ^Ad^
LBG 0.5%	0.773 ± 0.002 ^Ae^	0.767 ± 0.001 ^Ae^	0.638 ± 0.001 ^Ae^	0.759 ± 0.001 ^Ae^	0.904 ± 0.001 ^Ee^	0.747 ± 0.001 ^Fe^
LBGH 0.5%	0.755 ± 0.001 ^Af^	0.723 ± 0.001 ^Af^	0.716 ± 0.001 ^Cf^	0.817 ± 0.003 ^Cf^	0.505 ± 0.001 ^Ef^	0.537 ± 0.003 ^Ff^

Values are mean ± standard deviation from three repeated determinations. ^a, b, c, d, e, f^—means with different lowercase letters in the same column are significantly different at *p* < 0.05. ^A, B, C, D, E, F^—means with different uppercase letters in the same row are significantly different at *p* < 0.05. P 0.4%—Pectin 0.4%; PH 0.4%—Pectin hydrated 0.4%; GG—Guar gum 0.5%; GGH—Guar gum hydrated 0.5%; LBG 0.5%—Locust bean gum 0.5%; LBGH 0.5%—Locus bean gum hydrated 0.5%.

**Table 8:** Comparison of nutritional values of selected seeds with cow’s milk and final composition of plant-based dispersion [g/100g].

Ingredients	Carbohydrates (g/100g DM)	Proteins (g/100g DM)	Fat (g/100g DM)	Reference
Sunflower	20.7	21.3	53.0	[11,37]
Beans	56.0	25.1	1.8	[38,39,40]
Peas	41.4	21.2	1.2	
Cow’s milk **	4.5–5.1	3.2–3.4	3.1–3.8	[22,41,42]

Composition based on content from Table 2 based on 100 g of dry mass. ** Recalculated as 100 g of total dry mass.

**Table 9:** Ingredients in the final plant-based dispersion formulation.

Ingredients	Content in Emulsions [%]
Sunflower	6.0
Beans	2.0
Peas	1.9
Sunflower oil	0.1

**Table 10:** Table summary.

Variants	Colour (L*)	Colour Stability (ΔE*)	EI Stability Index	Particle Size (R) **	Degree of Turbidity (O) **
P 0.4%	Medium light	Minimum differences	168 h	Medium volatility	Large volatility
PH 0.4%	Medium light	Perceived differences	48 h	Low volatility	Low volatility
GG 0.5%	Brightest	Minimum differences	48 h *	Medium volatility	Medium volatility
GGH 0.5%	Bright	Minimum differences	24 h	Minimum volatility	Minimum volatility
LBG 0.5%	Darker than others	Minimum differences	48 h	Large volatility	Medium volatility
LBGH 0.5%	Darkest	Large difference	24 h	Large volatility	Large volatility

* The highest initial and post 48 h stability. ** During two weeks of storage. P 0.4%—Pectin 0.4%; PH 0.4%—Pectin hydrated 0.4%; GG—Guar gum 0.5%; GGH—Guar gum hydrated 0.5%; LBG 0.5%—Locust bean gum 0.5%; LBGH 0.5%—Locus bean gum hydrated 0.5%.

## Data Availability

The data is contained within the manuscript.
